# 

**Published:** 1807-06

**Authors:** 


### NEW MEDICAL PUBLICATIONS.

Medical Reports of Cases and Experiments, with Observations chiefly derived from Hospital Practice; by Samuel Argent Bardsley, M D. 8vo. 8s. bds.

First Lines of the Practice of Surgery; being an elementary Work for Students, and a concise Book of Reference for Practitioners, with such plates as are essential to the subject; by Samuel Cooper. 12s. 8vo. bds.

Dialogues in Chemistry, intended for the Instruction and Entertainment of Young People, in which the first Principles of that Science are fully explained. To which are added, Questions and other Exercises for the Examination of Pupils; by the Rev. J. Joyce, author of Scientific Dialogues in 6 vols. 7s.

The Companion to the Scientific Dialogues; or, Pupils Manual in Natural and Experimental Philosophy; containing a complete Set of Questions and other Exercises for the Examination of Pupils in the 6 vols of the Scientific Dialogues. To which is added, a Compendium of the principal Facts under each Department of Science; by the Rev. J. Joyce. 2s.

---

### To CORRESPONDENTS.

The enclosure of Mr. Shearly's paper, on which his name was written, having been mislaid, occasioned the omission of his signature to the cases of Aneurism and Mollities Ossium, in page 312.

The plate relating to Mr. Nosworthy's Case of Monstrosity, is unavoidably deferred till our next Number.

---

END OF VOL. XVII.

### INDEX.

A.

ABDOMEN, cafes of preternatural pulfation in the 188
Abernethy's Mr. cafe of femoral aneurifm 190
Abfcefs of the liver, cafe of 500
Acid, on the ufe of nitric 17
Adams, Dr. on epilepfy 390
——— on morbid poifons 567
Affufion, cold, its efficacy in ftrangulated hernia 55
Agues, on oxygen in 447
Albers's, Dr. cafe of preternatural pulfation 188
Albumen, experiments on 198
Alder, Mr. on contagion and fever 505
Alkali, ufe of volatile 293
——— effects of electricity on fixed 93
——— in tic douloureux, its ufe 388
Allen's, Mr. lectures announced 199
Amaurofis, remedy for 74
America, on the climate of 120
Amputation of the hip joint, on the 469
Amputation, obfervations on 537
Anæmia, obfervations on 472
Anderfon, Dr. on the guinea worm 171
———'s, Dr. letter to Mr. Ring 540
Andrews's, Dr. cafes of varicofe veins 297
——— cafe of popliteal aneurifm 302
Anemone, botanical defcription of the 73
Aneurifm, cafe of 5
——— cafe of femoral 190
——— on Dr. Cayley's cafe of 435
——— cafes of popliteal 302, 306
Angina pectoris, cafe of 9
Anguftura bark, on the 428
Animals, on the pulfations of 93
Anthon Dr. on yellow fever 126
Anti-Jacobin Review, extracts from 152
Antimonial powder, effects of 551
Apoplexy, cafe of 423
Aqua Ammon. puræ, its efficacy in tic douloureux 579
Aqua tuffana, account of 255
Aqueous humour in the eyes, on evacuating of the 193
Arum, botanical defcription of 72
Arnold, Dr. on infanity 84
Arrowhead, botanical defcription of 466
Aromatics, on the exhibition of, in gaftritis 159
Arfenic, its ufe in pforiafis 253
Artery, on compreffing the 438
———, on a ruptured 521
Afcites, cafe of 209
Affafœtida, as a vermifuge, on 173
Affalini, Mr. on the plague 506
Aftrometer, account of a new 582
Afthmatic complaints, remedy for 73
Atkinfon, Mr. cafes of hernia 227
Atmofphere, on an impure 103, 449
——— its effects on the pulmonary fyftem 292
Azote, on the gafeous oxyde of 51

B.

Babington's, Dr. lectures announced 199
Badham's, Dr. lectures announced 94, 392
Baglivi on the Difeafes of Rome 122
Balmis, Dr. his voyage round the world, to communicate vaccine inoculation 12
Barlow, Mr. on carbonate of iron in cancer 325
Bathing, on fea, in hydrophobia 185, 223
Bees, effects of fumach on 487
Beet-root, method of extracting fugar from 197
Betony, defcription and virtues of 562
Bellamy's, Dr. anfwer to Mr. Chalmers 39
——— Mr. Dawfon 45
Berkeley, cafes of cow-pox at 11
Bertin's, Mr. defcription of a new fyphon 197
Bibliographia Medica, profpectus of the 393
Binns, Dr. on fcarlatina 470
Birds, on the iris of 412
Black vomit defcribed by Hippocrates 124
Bladder, obfervations on Dr. Bellamy's cafe of difeafed 34
———, on difeafes of the 386
Blair's, Mr. cafe of obftruction in the œfophagus 20
——— lectures announced 94
Blood, on the 93
Blowpipe, account of Dr. Wollafton's *ib.*
——— Abbe Melograni's 196
Blumenbach on the iris 404
Boerhaave on hydrophobia 185
BOOKS, CRITICAL ANALYSIS OF NEW MEDICAL — Obfervations on the principal Difeafes of the Eyes, tranflated from the Italian of A. Scarpa, by James Briggs, 79. Malvern Waters, being a Republication of Cafes, formerly collected by John Wall, M. D. 83. Obfervations on the Nature, Kinds, Caufes and Prevention of Infanity, by T. Arnold, M. D. 84. The Edinburgh Medical and Surgical Journal, 187, 469

Practical Treatife on the Powers of Canthandes internally, by John Roberton, 279. Oratio in Theat. Coll. Regal. Med. Londin. et Harveii inftituto, habita à Chr. Pemberton, M. D. 281. An Account of a new difcovered Membrane in the Human Eye, by S. Sawrey, 283. Cafes and Cures of Hydrophobia, 286. A Letter to the Editor of the Britifh Critic, occafioned by fome Remarks in that Review on a Book entitled Cafes of pulmonary Confumption treated with Uva Urfi, 288. Obfervations on the Effects of various Articles of the Materia Medica in the Cure of Lues Venerea, by J. Pearfon, 375. Treatife on the Varieties and Confequences of Ophthalmia, by A. Edmondfton, M. D. 379. An Effay on Ophthalmia, by Henry Reid, 385. Obfervations on the Humulus Lupulus of Linnæus, by A. Freake, 386. Obfervations on Urinary Gravel and Stone, by H. Johnfton, *ib.* Hiftory of the Mineral Waters at Butterby, near Durham, by W. R. Clanny, M. D. 479. Addrefs to the Profeffors of Phyfic and Surgery, propofing the Inftitution of a Society for inveftigating the Caufe, &c. of Hydrophobia, *ib.* Account of the Ophthalmia which has appeared in England fince the Return of the Britifh Army from Egypt, by John Vetch, M. D. *ib.* Obfervations on Morbid Poifons, Chronic and Acute, by Jofeph Adams, M. D. 567. Sketch of the Revolutions of Medical Science, by P. J. Cabanis, tranflated by Dr. Henderfon, 574

BOOKS, NEW MEDICAL, ANNOUNCED. — Dr Clutterbuck's Inquiry into the Seat and Nature of Fever, 95. Dr. Herdman's Treatife on the Management of Infants, *ib.* Dr Trotter's View of the Nervous Temperament, *ibid.* Mr. Lawrence's Tranflation of Blumenbach's Comparative Anatomy, 200. Dr. Bardfley's Selection of Medical Reports of Cafes derived from Hofpital Practice, *ibid.* Dr Kinglake's Strictures on Mr. Parkinfon's Obfervations on Gout, *ibid.* Dr. A. P. Wilfon's Effay on the Nature of Fever, *ibid.* Dr. Robertfon, on the Natural Hiftory of the Atmofphere, 296. Dr. Bree's Inquiry into difordered Refpiration, *ib.* Mr. Cooper's Firft Lines of the Practice of Surgery, 296, 392. Dr. Smith's Introduction to Botany, 296. Dr. C. Fothergill's Voyage to the Northern Ifles, 296. Dr. Walker's Effay on Vaccination, 487. Chaptal, on the Application of Chemiftry to the Arts, 583. Mr. Nicholfon's Chemical Dictionary, *ibid.* Dr. Adams on Vaccine Inoculation, *ibid.* Mr. Rowland's Cafes of Scirrhus and Cancer 584

Botanical Defcription of Britifh plants 68, 371, 464, 558
Bougies, on Metallic Flexible 460
Boullam Fever, on the 128
Bourne, Dr. Reply to the Britifh Critic 288
Boys, Dr. Vindication of Navy Surgeons 251
Brain, on a demonftration of the 539
Breflaw, Regulations refpecting Vaccination at 58
Brewfter's, Mr. new aftrometer 582
Briggs, Mr. on difeafes of the eyes 79
Britifh Critic, anfwer to the 288
——— plants, botanical defcription of, 68, 371, 464, 558
Brookes's, Mr. lectures announced 94
Brown, a good dye for 69
Browne, John, furgeon to Charles II. account of 477
Burns, on the treatment of, 1, 21, 130, 132, 320, 321, 440
Burns, Mr. on crural hernia 23
——— on the cow-pox 471

C.

Cabanis Dr. on the revolutions of medical fcience 574
Cachexia fyphiloidea, on 377
Cahalot, defcription of the 483
Calculus, remarkable cafe of 64
Calomel, as a vermifuge, on 452
Camphor obtained from pepper-mint 560
Conception, remarkable cafes of 492
Convulfions, cafe of 548
Cancer, on carbonate of iron in, 201, 325
Cantharides, on the powers of 279
Canvas, method of preparing it for tubes 198
Caoutchouc, on diffolving of 95, 454
Carmichael, Mr. on carbonate of iron in cancer 201, 325
Carpue's, Mr. lectures announced 487
Carradori, Dr. on clearing water of oxygen 197
Cataract, obfervations on 81
Catheters, on metallic flexible 460
Cauftic in aneurifm, on 437
Cayley, Dr. on aneurifm 5
——— obfervations on the circular letter of 240
——— anfwer to 435
Cephalalgia, remedy for 469
Cephalica fympathetica, remedy for 562
Ceruffa acetata, its ufe in burns 21
Ceylon, vaccination at 517
Chalmers, Mr. anfwer to 39
——— on pneumonia 158
Chemical effects of electricity 93
Chevalier's, Mr. lectures announced 199
Children, on the treatment of 291

Children, on the puriform ophthalmia in 471
China, vaccine inoculation in 14
Ching's worm lozenges, fatal effects of 173
Chifholm, Dr. on yellow fever 128
Chlorotic cafes, fpecific for 468
Chriflie, Mr. on vaccination 517
Chyle, obfervations on 509
Clanny, Dr. on the mineral waters at Butterby, near Durham 479
Clarke, Dr. his correfpondence with the Jennerian Society 368
Clark's, Dr. lectures announced 94, 296
Clayton, Mr. on worm lozenges 173
Cleghorn, Dr. on yellow fever 125
Cline's, Mr. lectures announced 199
Clouds, on the formation of 485
Coal mine, fingular difeafe in a 472
———, method of conveying air into a 199
Cold affufion, its effects in hernia 55
——— in burns 133, 370, 440
Collins's, Mr. cafe of difeafed kidney 181
Confumption, on 79
——— on the lichen iflandicus in 255
Convulfions, cafe of 548
Contagion, obfervations on 101
——— and fever, on 505
Cook, Mr. on nitric acid 189
Cooper's, Mr. lectures announced 199
Cornea, on puncturing the 81
Correfpondents, to 95, 200, 296, 392, 488, 584
Cow-pox, cafes of at Berkeley 11
——— obfervations on 471
——— fee Vaccination
Cows, pulfation of 93
Crawford, Dr. on poke root 294
Crocodile, defcription of the 485
Crural hernia, ftructure of parts in 23
Curry's, Dr. lectures announced 199
Cyprus powder, compofition of 73

D.

Damp linen, obfervations on 84

Davis, Dr. on an hofpital for incurables 361

Davy, Mr. on fixed alkali 93

———, on fairy rings 197

———, on the influence of electricity 198

Dawfon's, Mr. anfwer to Mr. Thomas 258

———, reply to 45, 528

Death, cafe of fudden 188

De Carro, Dr. on the Guinea worm 173

Denmark, ftate of vaccination in 295

Denn fon s, Dr. lectures announced 94

Dewees, Dr. on fuperfœtation 489

Difpan, M. on gafeous oxyde of azote 51

Difeafe at New York, report on the 97

Difeafes and Cafualties in London, for 1806 96

——— in London, from Nov. 20, to Dec. 20, 1806 74, 77

——— from Dec. 20, 1806, to Jan. 20, 1807 183, 6

——— Jan. 20, to Feb. 20 289, 292

Difeafes in London Feb. 20, to March 20 387, 9

——— March 20, to April 20 482

——— April 20, to May 20 579, 581

———, on the application of galvanifm in 190

Dogs, on the madnefs of 184, 286

Douglas's, Mr cafe of convulfions 548

Dranunculus, defcription of the 171

Dropfical cafes, prevalence of 75

Dubois, Mr. on the Guinea worm 167

Duverney, on the iris 403

E.

Earneft's Mr. cafe of lepra 313
Eaft Indies, ftate of vaccination in 540
Ebullition, obfervations on 197
Edinburgh College of Phyficians elect Dr. Jenner an honorary fellow 139
——— Reviewers, remarks on 153
——— Medical and Surgical Journal reviewed 187, 469
——— college of furgeons, letter of 195
Edmonfton, Dr. on ophthalmia 317, 379
Effufion in the head, cafes of 551
Elaftic gum, how to diffolve 95, 454
Electricity, on the chemical effects of 93
———, on medical 267
———, influence of 198
Elephantiafis, on nitric acid in 189
Ellicott's Mr. voyage down the Ohio 127
Empiricifm, on 195
Epidermis, on the 507
Epilepfy, obfervations on 390
Errhine, defcription of a good 563
Exhalations, on peftilential 99
Experiments on gafeous oxide of azote 51
———, portable blow pipe for 93
———, on fifhes 197
———, galvanic 295
Eye, cafe of extirpation of the 187
———, new membrane in the human 283, 527
———, obfervations on the 486
Eyes, on difeafes of the 79
———, Dr. Walker on the 522
———, remedy for the 74
———, on evacuating the aqueous humour in inflammations of the 193

F.

Fairy rings, caufe of 197
Farifh, Mr his cafe of calculus 64
Farquharfon's Dr. letter to Dr. Harrifon 195
Femoral aneurifm, cafe of 190
Fever, efficacy of yeft in 503
Fever, (yellow) report on the 97
———, antiquity of the 124
———, in Minorca 125
———, on contagion and 505
———, on the caufe of 451
Fifhes, obfervations on 197
Fiftula lacrymalis, on the 283
Florence, ftate of vaccination at 63

### INDEX.

Fœtus, cafe of fmall-pox in the 470
—— defcription of a fingular 535
Foramen ovale, cafe of non-clofure of the 78
Fothergill's, Dr. reports of difeafes 74, 183, 289, 387, 482, 579
Foundling hofpital, vaccination at the 488
Fox's, Mr. lectures announced 199
Frampton's, Mr. lectures announced 94
Freake, Mr. on the gout 386
Friefe, Dr. on vaccination 57

G.

Gall, Dr. declines in reputation 295

Galvanic experiments 295

Galvanifm, its effects on difeafes 190

Gas, fatal effects of feptic 225

Gafeous oxide of azote, on 51

Gaftritis, exhibition of aromatics in 159

Germander, defcription and ufes of 466

Gibfon, Mr. on opthalmia in children 471

Girdleftone, Dr. on tapping at the navel 351

Gleet, ufe of cantharides in 280

——, cafe of obftinate 425

Golding's, Mr cafe of fmall-pox 514

Gonorrhœa, on injections in 547

Gout, effects of the Malvern waters in 83

——, ufe of humulus lupulus in 386

——, ufe of germander in 466

Grafswrack, defcription of 71

Gravel, on urinary 386

Ground ivy, defcription and virtues of 561

—— pine, defcription of 466

Guinea worm, on the 167

Gum, elaftic, how to diffolve 55, 454

Gunning's, Mr. lectures announced 94

Guy's hofpital, lectures at 199

H.

Harvey's cafes of conception 492
Hæmorrhage, ufe of leaves of the burnet in 70
Haighton's, Dr. lectures announced 199
Hall, Dr. on nitric acid 17
—— on burns and fcalds 132
—— Mr. on hydrophobia 328
Haller, on the iris 403
Hare-lip, caution in the operation for the 486
Harrifon, Dr. letter to 195
Harrup, Mr. on burns and fcalds 21
——, on elaftic gum 454
Harveian oration 281
Hauy, M on galvanic phenomena 295
Head, cafes of effufion into the 551
Head-ache, remedy for the 562
Headington's, Mr. lectures announced 94
Helena, introduction of the vaccine inoculation at St. 14
Hellebore, defcription and ufes of 375, 464
Henderfon, Dr. on feptic gas 225
Heifter, on the emollient practice 131
Hermftadt, Mr. on extracting fugar from the beet root 197
Hernia, on crural 23
—— cafe of ftrangulated 55
—— cafe of fcrotal 227, 430
Hicks, Mr. on hydrophobia 272
—— cafe of trifmus 277
Hill, Mr. R on vaccination 17[illegible], 329, 456
—— Mr. G. on medical reform 28
Hip joint, on the amputation of the 469
Hippocrates on fever 124
Hooping cough, obfervations on the 293
Horehound, defcription of white 564
Horfes, on the pulfation of 93
Hofpital for incurables, propofed 361
Huggan, Dr. on tapping at the navel 209
Hugo, Mr. on vaccination 365
Humulus lupulus, on the 386
Hunter, Dr. on yellow fever 120
Hydrocephalus internus, cafe of 188
—— cafe of acute 291
Hydrophobia, Dr. Fothergill on 183
——, Mr. Hicks on 272
——, M. Sabatier's cafes of 218, 344
——, cafes and cures of 286
——, ufe of volatile alkali in 293
——, efficacy of poke root in 294
——, Mr. Hall on 328
——, Mr. Ward on 353, 457
——, Mr. Soden on 432
——, addrefs on 479
Hydrothorax, cafe of 75
Hypopion, obfervations on 80

I.

Ichneumon fly, defcription of the 172

Icterical affections, on nitric acid in 17

Incurables, on an hofpital for 361

Indigo, preparation of a folution of 583

India, on the fcorpions of 170

Infantile difeafes, on 539

Inflammation after vaccination, on 156

Injections, obfervations on 547

Inoculation, hiftory of 146, 256

—— on fmall-pox 512

Infanity, obfervations on 84

Invifible girl, fecret of the 199

Iris, on the organization of the 403

Iron, effects of electricity on carbonate of 198

—— in cancer on 201, 325

Jamaica, progrefs of vaccination in 61

Jenner, Dr. his cafes of cow-pox 11

—— Dr. fubfcription in the Eaft for 540

—— Dr. Spalding's letter to 62

——, Dr. elected fellow of the college of phyficians at Edinburgh 139

Johnfon, Mr. on gravel and ftone 386

K.

Kaletchy, an Indian plant, account of 170
Kali fulph. its efficacy in lepra 313
Kentifh, Dr. on burns and fcalds 131, 440
Kidney, cafe of difeafed 181

Kinglake, Dr. on the theory of 21
——— on the anguftura bark 428
Knight, Mr. Copleyan medal prefented to 196
Knowles, Dr. his correfpondence with Dr. Clarke 368
Kuhn, Dr. on hydrocephalus internus 188

L.

Laird's, Dr. cafe of fmall-pox in the fœtus 470
Lanfdown's, Mr. method of making canvas tubes 198
Lancifi, on fevers 123
Larkfpur, (wild) defcription of the 71
Laffus, Mr. his cafe of poifon 422
Lectures announced:—*London Hofpital*, Mr. Headington and Mr. Frampton on Anatomy, Phyfiology, and Surgery, 94. Dr. Dennifon on Midwifery, *ib.* Mr. Brookes on Surgery, &c. 94 Mr. Wilfon on Anatomy, Phyfiology, and Surgery, *ib.* Dr. Badham on the Practice of Phyfic, Chemiftry, and the Materia Medica, 94, 342. Mr. Blair on Anthropology, *ib.* Dr. Clarke and Mr. Clarke on Midwifery, 94. Mr. Gunning on Surgery, 95. Mr. Moor on the Teeth, 95, 200. Mr. Taunton on Anatomy, Phyfiology, and Surgery, 95, 487. *St. Thomas's and Guy's Hofpitals*, viz. Meffrs. Cline and Cooper on Anatomy and Surgery, Drs. Babington and Curry on the Practice of Medicine, &c. Dr. Babington and Allen on Chemiftry, Mr. Allen on Experimental Philofophy, Dr. Haighton on Phyfiology and Midwifery, Mr. Fox on Odontology, 199. Mr. Chevalier on Surgery, *ib.* Dr. Reid on Medicine, 200, 392 Mr. Milburne on Surgery, 200. Mr. Carpue on Anatomy and Surgery, 487.
Le Grange, M. on tannin 392
Lepra, cafe of 313
Leprous eruption, cured 69
Lichen iflandicus, on the 255
Ligatures, on 193
Lightning, cafe of the effects of 50
Lime-tree, defcription of the 69
Lipetzk, analyfis of the waters of 486
Liquors, on the abufe of fpirituous 75
Little, Mr. on ftrangulated hernia 55
Liver, cafe of abcefs of the 500
Liver, on the functions of the 508
London, difeafes and cafualties in 76
——— hofpital, lectures at 94
——— medical fociety, proceedings of the 198, 390
Lowndes, Mr. on medical electricity 267
Lues venerea, on the cure of 375
Lungs, on lichen iflandicus in confumptions of the 255
Lyons, Dr. on the lichen iflandicus 255

M.

Madeira, accommodations in 391
Madnefs, obfervations on 89
Madrafs, ftate of vaccination at 544
M'Dowell, Dr on burns and fcalds 320
Malvern waters, account of 83
Mammalia, difcove y of a new animal o the order of 583
Marjoram, defcription and ufes of the wild 565
Marfh marigold, defcription of the 465
——— miafmata, on the fevers of 447
Marfon, Mr. on burns and fcalds 130
——— his anfwer to Mr. Ring 136
——— reply to 239
Maton, Dr. on the humulus lupulus 386
Maunoir, Mr. on the iris 403
Mead, Dr his famous powder 185
Medical reform, obfervations on 28
——— theories on 66
——— and phyfical intelligence 93, 196, 293, 390, 485, 582
——— publications, new 96, 200, 296, 488, 584
——— electricity, on 267
——— art, profpectus of a hiftory of the 393
Medical fcience, on the revolutions of 575
Melograni's blow pipe, defcribed 196
Mercury, its ufes in lues venerea 376
——— its effects on the liver 511
Metallic bougies, account of the flexible 461
Milburne's, Mr. lectures announced 200
Miller, Dr. on the malignant difeafes of New York 97
Mineral kingdom, effects of electricity on the 198
Mines, on explofions from 5
——— fingular difeafe in 472
Minorca, yellow fever indigenous in 125
Mollities offium, cafe of 311
Mongiardini, Dr. on galvanifm 190
Montrofs's, Dr. cafe of rheumatic fuppuration 217
Morbific, on the ufe of the word 568
Morgan's, Mr. cafes of vaccination 312
——— Dr. obfervations on purgatives 470
Moor's, Mr. lectures announced 94, 204
Mofeley, Dr. account of 61
——— anfwer to 175, 329
Mudd, Mr. on extirpation of the eye 187
Mufcular motion, on 93

N.

Navel, on tapping at the 209, 351
Navy furgeons, vindication of 30, 250
New York, account of the malignant difeafe at. 97
Nitrous gas, fudden death from 188
Nofworthy's, Mr. defcription of a remarkable fœtus 533

O.

Oesophagus, case of obstruction in the 20

Ohio, voyage down the 127

Oil, surgical instruments to be dipped in 198

Ophthalmia, observations on epidemic 193

——— Dr. Edmondston on 317, 379

——— Mr. Reid on 385

——— in children on 471

——— Dr. Vetch on 479

Opium, in tetanus on 211

——— case of poison by 422

Ormskirk remedy, on the 185

Oxide of azote, on gaseous 51

Oxygen, on freeing water from 197

——— in agues, on 447

P.

Paralysis, case of 427

Paralytic attacks, frequency of 483

Pascalis, Dr. on abscess of the liver 500

Pasque flower, description of 371

Pearson, Mr. on the lues venerea 375

Pemberton's, Dr. Harveian oration 281

Pennyroyal, description of 561

Pepper-mint, on the essence of 560

Percival's, Dr. method of treating hydrophobia 328

Peripneumony, case of 390

Pierson, Mr. on the pulsations of animals 93

Pile wort, description of the common 372

Plague, on the 450, 506

Plants, description of British 68, 371, 464, 558

Pneumonia, on 158

Poison, case of by opium 422

———, use of alkali against 293

Poisons, remedy for 565

Poke root, efficacy of 294

Popliteal aneurism, cases of 302, 306

Porterfield, on the iris 403

Portsmouth (New Hampshire) bill of mortality at 78

Pott, Mr. on the treatment of burns 131

Preternatural pulsation of the abdomen, case of 188

Procedentia iridis, on 81

Pseudo syphilis, case of 229

——— observations on 535

Psoriasis, on arsenic in 253

Pterygium, observations on 80

Pulmonary complaints, on 292

——— remedy for 562

Pulsations of animals, on the 93

Pupil, on the artificial 82, 403

Purgatives, on the administration of 470

Q.

Quadrupeds, on the iris in 413
Quarantine, on the laws of 118, 506
Questions for the Fothergillian prize medal 198

R.

Ranunculus, description of the 372

Rattle snake, singular effects of the bite of a 295

Reed, Mr. on vaccination 248

Reform, on medical 28

Reid's, Dr. lectures announced 200, 394

——— Mr. on ophthalmia 385

Rheumatic suppuration, case of 257

Rheumatism, remedy for 72

——— on electricity in 267

Ring's, Mr. case of angina pectoris 9

——— cases of cow-pox 11

——— on vaccination 57, 140, 233, 540

——— cases of secondary small-pox 137

Roberton, Mr. on cantharides 279

Rogers's, Mr. case of the effects of lightning 50

Rome, on the climate and diseases of 122

Rooms, improved stove for heating 583

Royal touch, on the 477

Royston's, Mr. prospectus of a Bibliographia Medicæ 393

Ruptured poor, observations on the 584

Rush, Dr. on the theory of 161

Russel, Dr. on the plague 507

Russia, effects of lightning in 50

Rue wood, description of meadow 372

S.

Sabatier's, Mr. cases of hydrophobia 218, 344

Sacco, Dr. his success in vaccine inoculation 63

Salisbury plain, herbage of 70

Samiel wind, account of the 449

Saumarez, Mr. on the theory of 163

Sawrey, Mr. on fistula lacrymalis 283, 527

Scalds, on the treatment of 1, 21, 130, 132, 320, 440

Scarpa, Prof. on diseases of the eyes 79

Scarlatina, on the cure of 470

Scarlatina anginosa, on 186

Scorpions, remedy for the bite of 179

Scott's Mr. case of hernia 430

Scrofula, on the royal touch for the 477

Scrotal hernia, case of 430

Seguin, Mr. on albumen 198

Septic gas, fatal effects of 225

Silesia, progress of vaccination in 58

Small-pox, cases of secondary 137, 147, 546

——— in the foetus, case of 470

———, impropriety and danger of inoculating for the 148, 512

———, case of, after vaccination 514

Smart's Mr. case of inflammation after vaccination 156

Smyth, Mr. on flexible bougies and catheters 469

Soden, Mr. on hydrophobia 432

Somerville, Mr. on fumach 487
Spain, progrefs of vaccination in the provinces of 12
Spalding, Dr. on the cow pox 62
——— his account of difeafes 78
——— cafe of apoplexy 423
Spearmint, defcription and virtues of 558
Spearwort, defcription of 372
Spens, Dr. his letter to Dr. Jenner 139
Spirituous liquors, abufe of 75
Spleen, on the ufe of the 160
Squinting, obfervations on 522
Squire's Dr. lectures announced 94
Stoker, Dr. on antimonial powder 551
Stone, on the gravel, &c. 386
Stove for heating rooms, a new 583
Strabifm, obfervations on 523
Stuart's Dr. letter to Dr. Jenner 142
Sugar from beet root, how to extract 197
Sumach, its effects on bees 487
Superfœtation, effay on 489
Surgical inftruments to be dipped in oil 198
Swan, on the iris of the 416
Sydenham on the treatment of burns 131
——— character of 282
Sympathy, on the doctrine of 211
Syphilis, on the ufe of 476
——— cafe of pfeudo 229
——— obfervations on 536
Syphon, defcription of a new 197
Tabes mefenterica, cafe of 76
Tannin, on the nature of 392
Tapping at the navel, on 209, 351
Taunton's Mr. lectures announced 94, 487
——— Mr. on the ruptured poor 584
Tetanus cafe of 211
Theories, on medical 66
Thomas's St. Hofpital, lectures at 199
Thomas, Mr. on a cafe of difeafed bladder 34
——— anfwer to 258
——— reply to Mr. Dawfon 528
Thyme, defcription of common 566
Tic doulouroux, cafe of 387
——— remedy for 579
Tilia Americana, its efficacy in fcalds 321
Tinea, cafe of 187
Tobacco glyfter, its efficacy in an obftruction 20
——— compofition of herb 563
Tracy's Dr. cafe of tetanus 211
Trifmus, cafe of 277
Tubes flexible, for coal mines 198
Turpentine, on its ufe in burns 4, 22, 131, 445
Typhus, prevalence of 77
Typhus fever, efficacy of yeft in 503

U.

Urinary gravel and ftone, obfervations on 386

Vaccination, progrefs of in Spain 12
——— Mr. Verral's cafes of 25
——— Dr. Friefe on 57
——— Mr. Wefton, on 61
——— Mr. Ring, on 62, 233
——— Dr. Spalding, on 62
——— Mr. Wake, on 95
——— Mr. Marfon, on 136
——— cafe of inflammation after 156
——— Rev. R. Hill, on 175, 329, 456
——— Mr. Reed, on 248
——— in Denmark, progrefs of 295
——— cafes, of 312
——— Mr. Hugo, on 365
——— at the Foundling Hofpital 488
——— Mr. Golding's cafe of 514
——— Mr. Chriftie, on 517
Varicofe veins, cafes of 297
Varley, Mr. on atmofpherical phenomena 485
Vegetable phyfiology, difcoveries in 196
Vegetables, albumen in 198
Veitch, Mr. on the amputation of the hip joint 469
Vermifuge, on calomel as a 452
———, ufe of hellebore as a 464
Verral, Mr. on vaccination 25
Vervain, defcription and ufes of 468
Veficle, on the rupture of the vaccine 136
Vetch, Dr. on ophthalmia 479
——— remarks on 317
Volney on the yellow fever 120

W.

Wake, Mr. on Vaccination 95
Wake-Robin, defcription of 72
Walker, Dr on the fpleen 160
——— on the eye 522
Wall, Dr. on the Malvern Waters 83
Ward, Mr. on hydrophobia 353, 457
Wardrop, Mr. on the inflammation of the eyes 193
Water, benefit of cold, in burns 5
——— on freeing it from oxygen 197
——— defcription of a new fyphon for raifing 197
——— lilly, defcription of 68
——— on the treatment of burns by cold 320, 440
Watkins, Dr. on Yellow fever 127
——— Mr. on yeft in fever 503
Wefton, Mr. on vaccination 61
Willan, Dr, on vaccination 150
Willey, Dr. on the hooping cough 293
Williams, Mr. on the liver 508
Wilfon's Mr lectures announced 94
Wollafton's Dr. blow pipe defcribed 93
Worm lozenges, fatal effects of 173
——— medicines, on 452

Y.

Yellow fever, Dr. Miller on the 97

——— antiquity of 124

——— indigenous at Minorca 125

——— Dr. Anthon, on 126

——— Mr. Ellicott, on 127

——— Dr. Watkins, on *ibid.*

Yellow fever, Dr. Chisholm, on 128

Yest, its efficacy in typhus fever 503

Young, Mr. on the iris 403

Z.

Zinn, on the motions of the iris *ib.*

---

### REFERENCES TO THE PLATES.

Mr. Young, on the Organization of the Iris - - - - - - 403
Mr. Nosworthy's Case of Monstrosity - - - - - - - 533

